# Endovascular Treatment of Nonsaccular Posterior Circulation Aneurysms: A Comparative Meta-analysis of Flow Diversion versus Stent-Assisted Coiling

**DOI:** 10.1055/a-2964-8885

**Published:** 2026-10-08

**Authors:** David Cho, Gerardo Duran, MacGregor Thomas, Abdurrahman F. Kharbat, Andrew M. Bauer, Christopher S. Graffeo, Michael J. Feldman

**Affiliations:** 1 Department of Neurosurgery 220831 University of OklahomaOklahoma CityOklahomaUnited States

**Keywords:** intracranial aneurysms, posterior circulation, flow diversion, stent-assisted coiling, fusiform aneurysm

## Abstract

**Background** Fusiform and giant aneurysms of the posterior circulation carry a dismal natural history. Flow diversion (FD) and stent-assisted coiling (SAC) are the two principal reconstructive endovascular strategies, yet no meta-analysis of direct comparative studies exists to guide selection between them.

**Methods** A Preferred Reporting Items for Systematic reviews and Meta-Analyses–compliant systematic review of MEDLINE and Embase (2011–2026) identified comparative studies of adult patients with nonsaccular posterior circulation aneurysms reporting outcomes following FD and SAC. Random-effects meta-analyses were performed with the meta package in R (v4.3.3). Two planned sensitivity analyses and leave-one-out analysis assessed robustness.

**Results** Five studies comprising 209 aneurysms (102 FD, 107 SAC) met inclusion criteria. No significant differences were observed between FD and SAC for favorable functional outcome, satisfactory aneurysm occlusion, or ischemic stroke. FD was associated with significantly higher all-cause mortality (odds ratio [OR]: 2.06; 95% confidence interval [CI]: 1.11–3.84; *p* = 0.03), aneurysm rupture (OR: 2.59; 95% CI: 1.15–5.82; *p* = 0.03), and in-stent thrombosis (OR: 8.15; 95% CI: 1.43–46.40; *p* = 0.03). All three findings demonstrated fragility on sensitivity and leave-one-out testing.

**Conclusion** FD and SAC produce comparable functional outcomes and occlusion rates for nonsaccular posterior circulation aneurysms. FD may carry higher mortality, rupture, and in-stent thrombosis, though these signals did not withstand sensitivity analyses and could not be separated from selection bias toward complex aneurysms. These findings support both modalities as viable reconstructive options while underscoring anatomy-driven treatment selection and the need for prospective validation.

## Introduction

Fusiform and giant aneurysms of the posterior circulation carry cumulative mortality rates that exceed those of virtually any other intracranial vascular lesion. 1 Open microsurgical access is constrained by brainstem perforator anatomy and narrow corridors, 2 driving an accelerating shift toward endovascular management. Consequently, endovascular treatment has become the preferred approach for the treatment of nonsaccular posterior circulation aneurysms. 3 4 5 6 7 8 9 10

Both flow diversion (FD) and stent-assisted coiling (SAC) are reconstructive endovascular approaches aimed to preserve the parent artery, but through different mechanisms: FD redirects flow away from the aneurysm sac through a low-porosity stent, promoting gradual thrombosis and neointimal remodeling, whereas SAC provides a scaffold across the aneurysm neck to retain coils. In saccular aneurysms, FD has demonstrated high occlusion rates and favorable neurological outcomes. 10 11 12 13 14 15 16 17 In nonsaccular posterior circulation morphologies, evidence is more limited with an incidence of <1% 18 ; a prior meta-analysis of single-arm FD series reported complete or near-complete occlusion in only 52% and favorable outcomes (modified Rankin scale [mRS] ≤ 2) in 31–71%. 19

SAC predates FD in this population by nearly a decade and offers the advantage of immediate aneurysm sac packing. 6 7 8 9 10 FD eliminates the need for intrasaccular access but introduces concerns of thromboembolism, potentially due to the increased resistance to perforator artery filling caused by the increased metal surface area. 20 Despite these conceptual differences, no pooled analysis of head-to-head studies exists. This meta-analysis provides the first direct comparative evidence between FD and SAC for nonsaccular posterior circulation aneurysms.

## Methods

### Search Strategy

We conducted a Preferred Reporting Items for Systematic reviews and Meta-Analyses–compliant systematic review of MEDLINE and Embase from January 2011 through February 2026 ( Fig. 1 ). A supplemental bibliography review in Google Scholar was also performed to screen for additional candidate citations not captured by the primary screen. Search terms included: nonsaccular, dolichoectatic, fusiform, giant, large, dissecting, SCA, AICA, cerebellar, PCA, PICA, vertebral, basilar, vertebrobasilar, flow diversion, silk, pipeline, fred, surpass, stent-assisted coiling, and endovascular. Three reviewers independently screened abstracts and full texts. Studies were included if they enrolled adult patients with nonsaccular aneurysms (fusiform, giant, or dolichoectatic) in the vertebrobasilar or posterior cerebral artery territories with extractable comparative data for FD versus SAC. Studies limited to vertebral artery dissecting aneurysms were excluded as a morphologically distinct entity. Of 685 candidate records, 547 unique abstracts were screened; 34 underwent full-text review, yielding five studies.

**Fig. 1:**
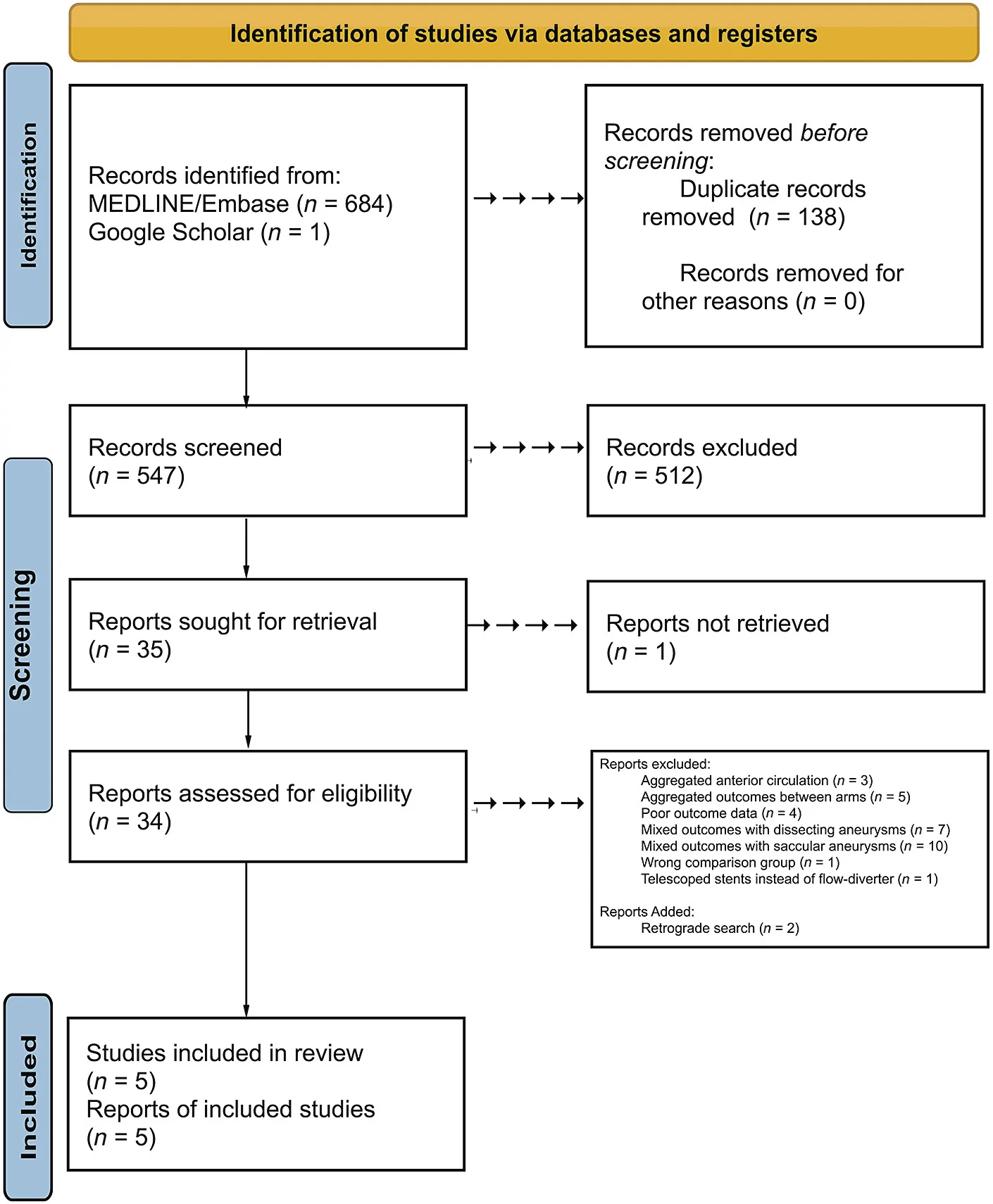
Flowchart outlining the systematic review process per PRISMA protocol. PRISMA, Preferred Reporting Items for Systematic reviews and Meta-Analyses.

### Outcomes

The primary outcome was favorable functional status (mRS: 0–2) at latest follow-up. Secondary outcomes included satisfactory aneurysm occlusion (Raymond–Roy grade I–II), complete occlusion (Raymond–Roy grade I), in-stent thrombosis, ischemic stroke, aneurysm rupture, and all-cause mortality.

### Statistical Analysis

Random-effects meta-analyses were performed with the meta package (v8.1–0) in R (v4.3.3) using the inverse–variance method with restricted maximum-likelihood estimation of between-study variance and Hartung–Knapp adjustment. Odds ratios (OR) were used for rare complications, whereas risk ratios (RR) were used for proportion-based metrics. A continuity correction of 0.5 was applied to all four cells of every study to retain double-zero studies, per Cochrane rare-event methodology. Heterogeneity was assessed using the Cochran *Q* test and *I* ^2^ statistic.

Two prespecified sensitivity analyses were performed: the first excluding studies limited to vertebral artery aneurysms and the second excluding studies limited to basilar artery aneurysms. These served to probe whether the comparative safety profile of FD and SAC differs by anatomic territory, which is a clinically relevant question given the distinct perforator anatomy, hemodynamic environment, and technical demands of vertebral versus basilar interventions. Leave-one-out analysis was conducted for outcomes with three or more studies. Publication bias was assessed by funnel plot inspection.

## Results

### Study Characteristics

Five studies 4 6 7 9 21 encompassing 209 aneurysms (102 FD, 107 SAC) met inclusion criteria. Aneurysms were predominantly fusiform ( *n* = 188) and giant ( *n* = 53). Aneurysms were symptomatic in 131/195 (67.1%). The most common presenting symptoms were headache ( *n* = 36, 17.2%) and dizziness ( *n* = 34, 16.3%), followed by ischemic symptoms ( *n* = 26, 12.4%), subarachnoid hemorrhage ( *n* = 7, 3.3%), cranial nerve deficit ( *n* = 6, 2.9%), vomiting ( *n* = 6, 2.9%), sensory deficit ( *n* = 5, 2.4%), mass effect ( *n* = 3, 1.4%), and unspecified symptoms ( *n* = 7, 3.3%). Mean patient age ranged from 52.4 to 60.2 years; mean clinical follow-up was 14.1 months (7.9–21.0). The vertebral artery was the most common site ( *n* = 139), followed by the basilar trunk ( *n* = 53), vertebrobasilar junction ( *n* = 7), posterior cerebral artery ( *n* = 2), and basilar tip ( *n* = 2). Flow diverter stent type were the Pipeline Embolization Device (PED; *n* = 57), Tubridge ( *n* = 5), Silk ( *n* = 5), and undefined ( *n* = 23). Multiple stents were utilized via a double overlapped fashion ( *n* = 8), triple overlapped ( *n* = 2), and overlapped with Leo stents ( *n* = 2). Adjunct coiling for FD was present in a minority of cases ( *n* = 3). Dual antiplatelet therapy was specified in four of five studies: aspirin/clopidogrel ( *n* = 88), aspirin/prasugrel ( *n* = 6), aspirin/cilostazol ( *n* = 1), aspirin/clopidogrel/cilostazol ( *n* = 3), and undefined ( *n* = 4). Publication bias was not detected visually ( **Supplementary Fig. S1** [available in online version only]).

### Functional and Occlusion Outcomes

Favorable functional outcome did not differ between FD and SAC (RR: 1.02; 95% CI: 0.94–1.11; *p* = 0.48; *I* ^2^ = 0%; Fig. 2 ), nor did satisfactory aneurysm occlusion (RR: 1.03; 95% CI: 0.91–1.16; *p* = 0.56; *I* ^2^ = 0%; Fig. 3 ) or complete occlusion (RR: 1.22; 95% CI: 0.97–1.54; *I* ^2^ = 0%; Fig. 4 ). All three were stable across both sensitivity analyses and leave-one-out testing ( Figs. 2 3 4 , **Supplementary Fig. S2** [available in online version only]).

**Fig. 2:**
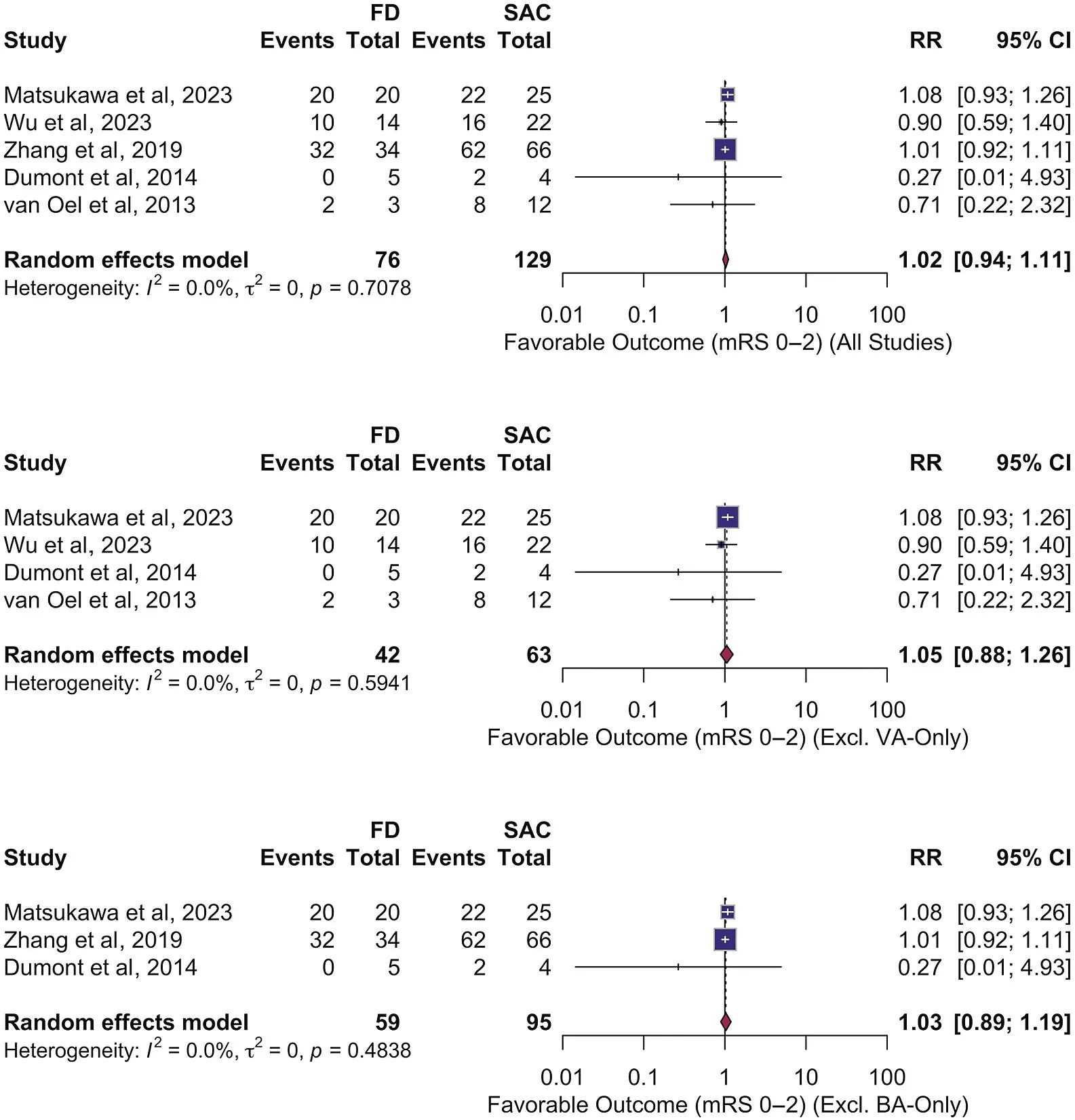
Forest plot depicting favorable functional outcome (mRS: 0–2) between flow diversion and stent-assisted coiling with sensitivity analyses excluding the vertebral artery–only cohort and the basilar artery–only cohort. CI, confidence interval; FD, flow diversion; mRS, modified Rankin scale; RR, risk ratio; SAC, stent-assisted coiling.

**Fig. 3:**
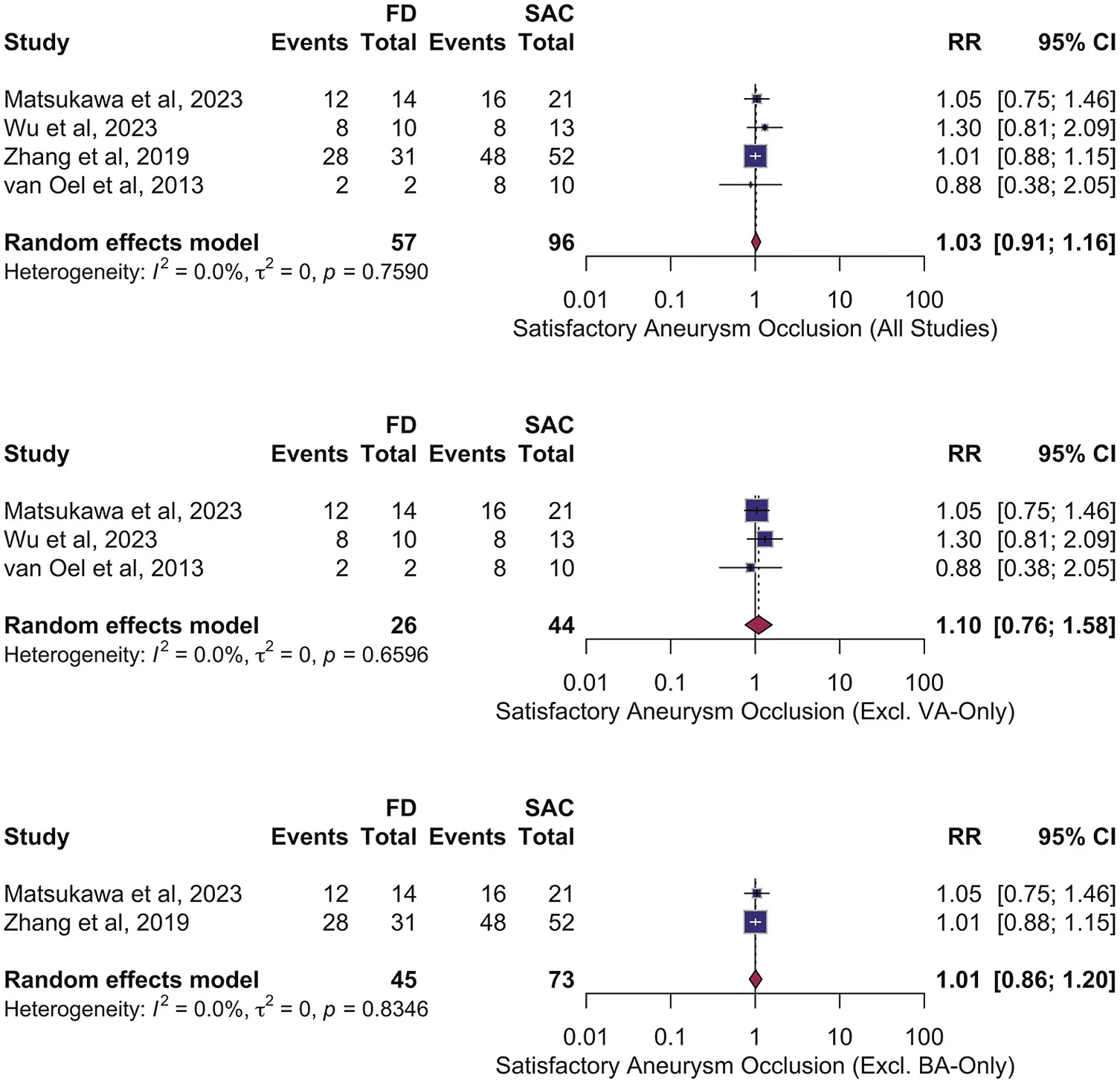
Forest plot depicting satisfactory aneurysm occlusion (Raymond–Roy grade I–II) between flow diversion and stent-assisted coiling with sensitivity analyses excluding the vertebral artery–only cohort and the basilar artery–only cohort. CI, confidence interval; FD, flow diversion; RR, risk ratio; SAC, stent-assisted coiling.

**Fig. 4:**
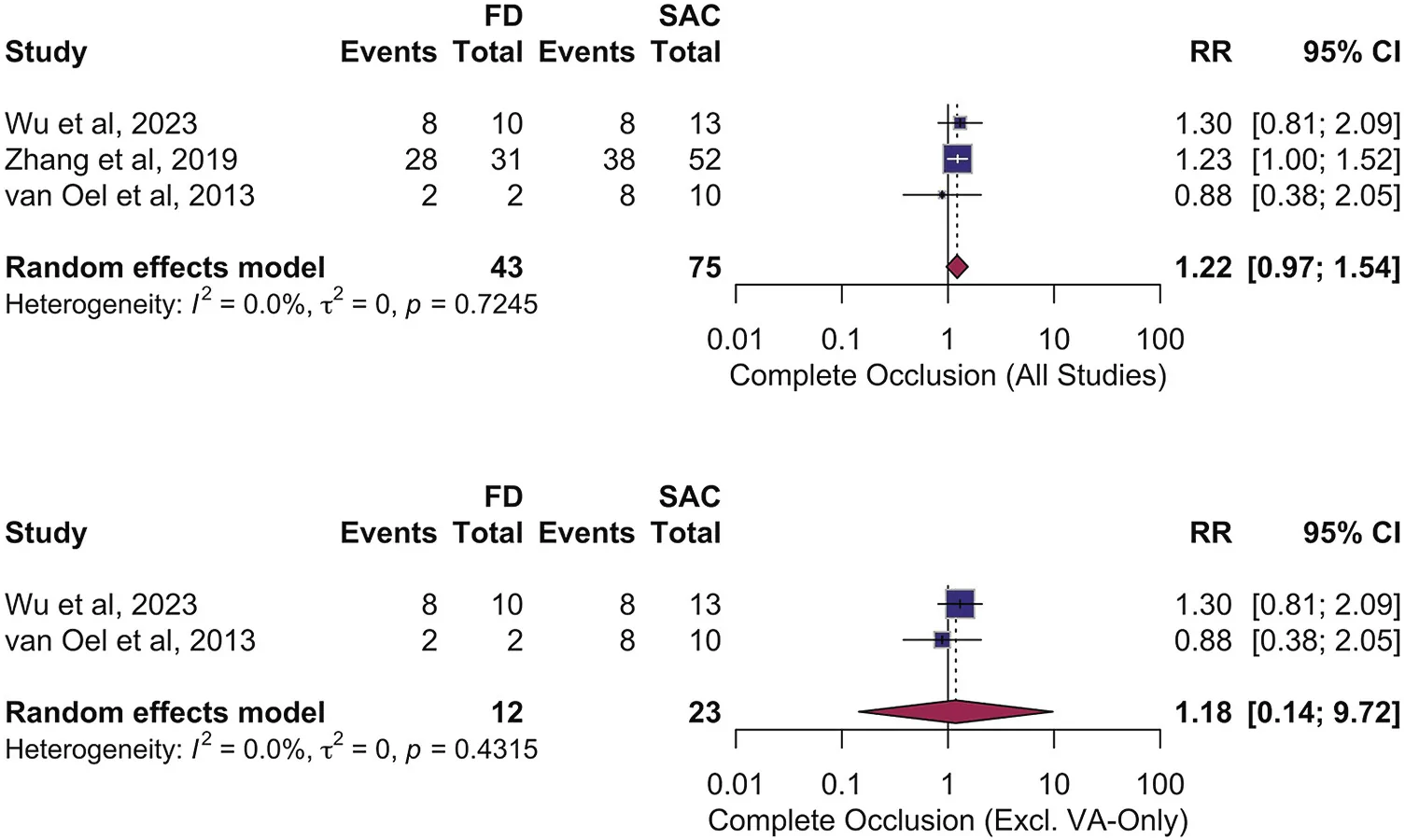
Forest plot depicting complete aneurysm occlusion (Raymond–Roy grade I) between flow diversion and stent-assisted coiling with sensitivity analyses excluding the vertebral artery–only cohort. CI, confidence interval; FD, flow diversion; RR, risk ratio; SAC, stent-assisted coiling.

### Postprocedural Complications

FD was associated with significantly higher aneurysm rupture (OR: 2.59; 95% CI: 1.15–5.82; *p* = 0.03; *I* ^2^ = 0%; Fig. 5 ). Neither sensitivity analysis preserved significance (excluding VA: *p* = 0.07; excluding BA: *p* = 0.08), and only one leave-one-out permutation maintained it ( Fig. 5 , **Supplementary Fig. S2** [available in online version only]). FD was not significantly different in rate of ischemic stroke than SAC (OR: 1.33; 95% CI: 0.39–4.48; *p* = 0.55; *I* ^2^ = 0%) across main, sensitivity, and leave-one-out analyses ( **Supplementary Figs. S2 and S3** [available in online version only]). However, FD was associated with significantly higher in-stent thrombosis (OR: 8.15; 95% CI: 1.43–46.40; *p* = 0.03; *I* ^2^ = 0%; **Supplementary Fig. S4** [available in online version only]). All contributing studies recorded zero events in the SAC arm. Neither sensitivity analysis preserved significance (excluding VA: *p* = 0.13; excluding BA: *p* = 0.37; **Supplementary Fig. S4** [available in online version only]), and only one of four leave-one-out permutations maintained it ( **Supplementary Fig. S2** [available in online version only]).

**Fig. 5:**
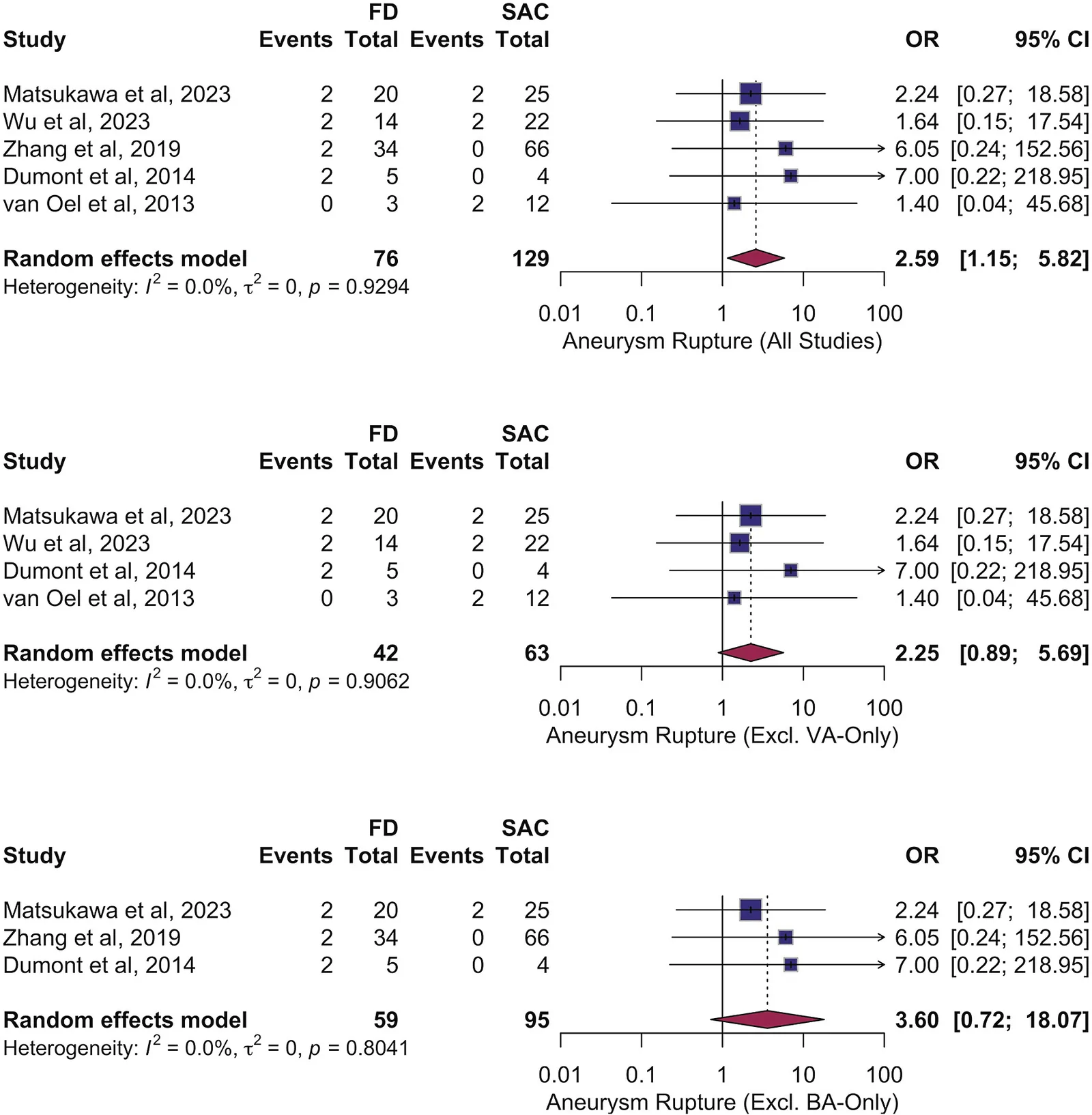
Forest plot depicting aneurysm rupture between flow diversion and stent-assisted coiling with sensitivity analyses excluding the vertebral artery–only cohort and the basilar artery–only cohort. CI, confidence interval; FD, flow diversion; RR, risk ratio; SAC, stent-assisted coiling.

### Mortality

FD was associated with significantly higher all-cause mortality (OR: 2.06; 95% CI: 1.11–3.84; *p* = 0.03; *I* ^2^ = 0%; Fig. 6 ). Excluding the basilar artery–only cohort preserved significance (OR: 2.60; 95% CI 1.13–5.98; *p* = 0.04), as opposed to excluding the vertebral artery–only cohort (OR: 2.07; 95% CI 0.88–4.86; *p* = 0.07; Fig. 6 ). On leave-one-out analysis, exclusion of one study 19 strengthened the association (OR: 2.86; *p* = 0.008), whereas exclusion of any other study yielded *p* -values between 0.07 and 0.10 ( **Supplementary Fig. S2** [available in online version only]).

**Fig. 6:**
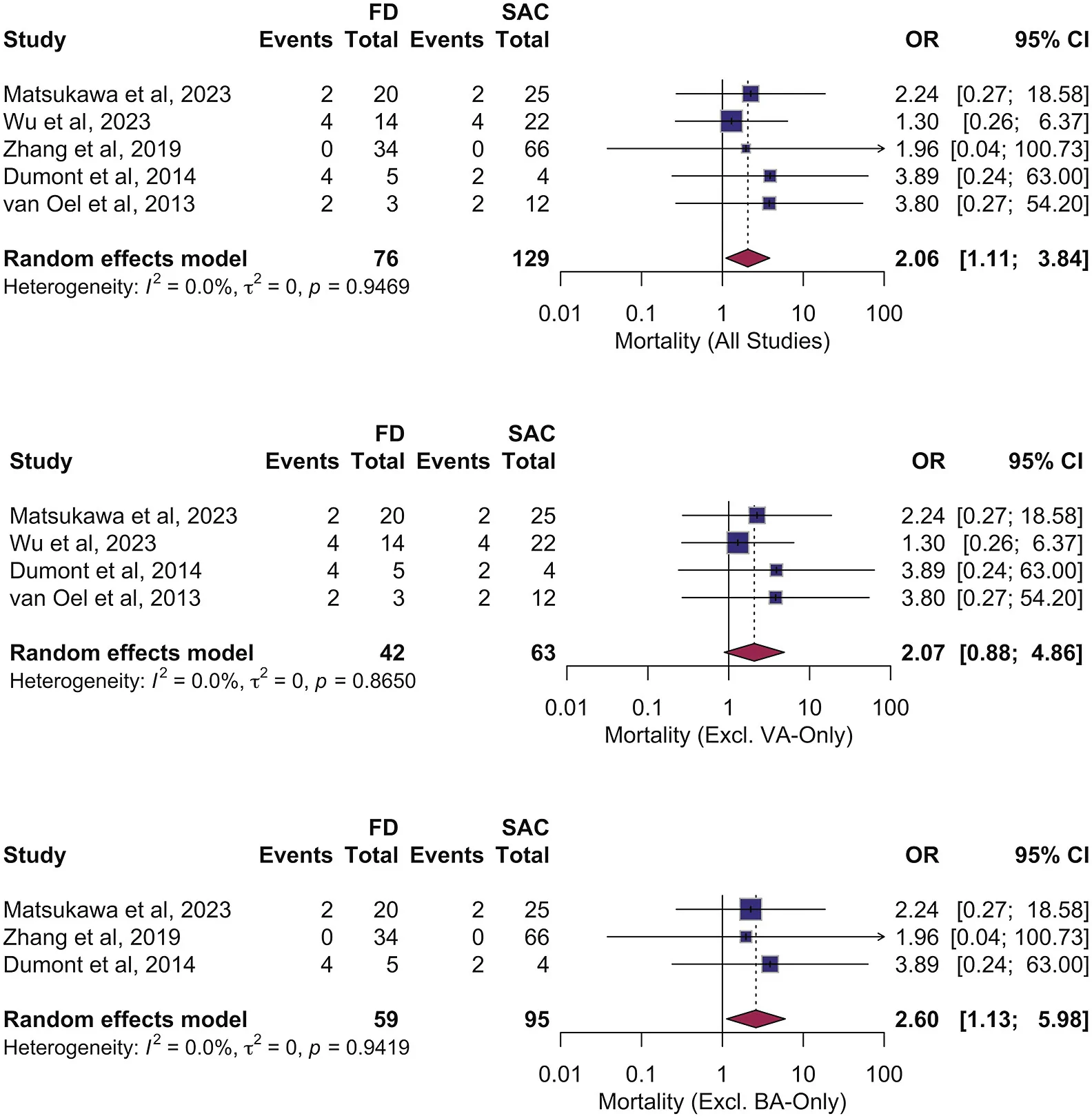
Forest plot depicting all-cause mortality between flow diversion and stent-assisted coiling with sensitivity analyses excluding the vertebral artery–only cohort and the basilar artery–only cohort. CI, confidence interval; FD, flow diversion; RR, risk ratio; SAC, stent-assisted coiling.

### Publication Bias

A funnel plot of included studies was conducted that showed no asymmetry visually ( **Supplementary Fig. S4** [available in online version only]).

## Discussion

This meta-analysis presents the first pooled direct comparison of FD and SAC for posterior circulation nonsaccular aneurysms. Our findings demonstrate therapeutic equipoise in functional outcomes and occlusion rates with an increased risk of several complications following flow-diverter deployment. FD was associated with higher rate of aneurysm rupture, stent thrombosis, and mortality. Notably, ischemic stroke was comparable between FD and SAC. Taken together, these results highlight comparable effectiveness of both endovascular strategies for fusiform and giant posterior circulation aneurysms while cautioning against FD deployment in certain scenarios.

### Postoperative Complications in Flow Diversion

A prior meta-analysis similarly observed fewer periprocedural complications with SAC, 22 though it included only one directly comparative study. Our findings reflected a similar pattern with the exception of rate of ischemic stroke being comparable between both strategies. Considering the nature of this comparative analysis of retrospective studies, this is likely explained by a selection bias preferring SAC in perforator dense segments, which protects patients receiving FD who would have otherwise suffered perforator stroke. The higher rate of aneurysm rupture and in-stent thrombosis observed following FD did not withstand significance when excluding basilar artery-only or vertebral artery-only studies. Aneurysm rupture may be mediated by a longer duration of antiplatelet therapy seen with FD, which also beckons careful consideration of FD in patients with medical conditions that increase risk of hemorrhage. On the other hand, prolonged antiplatelet regime is essential to prevent the increased risk of stent thrombosis seen in FD. Surveillance bias may also contribute, as stent thrombosis is a recognized and actively monitored complication of flow-diverter constructs. Regardless of the loss of significance in sensitivity analyses, higher postoperative complications seen after FD in this population highlight a potential safety advantage of SAC. Overall, this underscores the balancing act that is required between rigorous antiplatelet management and the patient’s overall risk profile for hemorrhage when deploying flow diverters in the posterior circulation for nonsaccular aneurysms.

The most significant finding is an increased risk of all-cause mortality following FD than SAC, which persisted, unlike other significant findings, when excluding basilar artery-only studies. There was minimal skew away from FD when excluding basilar artery-only studies (56/148, 37.8%) versus when excluding vertebral artery-only studies (38/97, 39.2%). It is likely that FD is reserved for complex aneurysms less suitable to SAC in the vertebral artery, which may carry a better prognosis than the same stratification in the basilar artery. Future data and studies may clearly affirm this increased risk though the overall mortality is significantly reduced with either treatment (17/195, 8.7%: FD: 9/71, 12.7%; SAC: 8/124, 6.5%) compared with the natural history of symptomatic fusiform aneurysms, which approaches 80% in 5 years. 23

### Ischemic Risk in Posterior Circulation Flow Diversion

Perforator territory infarction has been the central concern with posterior circulation FD since its introduction. Early outcomes in large fusiform vertebrobasilar aneurysms were comparable or inferior to best medical management 24 and open cerebrovascular surgery. 25 Contemporary data tell a different story. One series reported 11 of 12 patients recovering to mRS 0 to 1 with a single perforator infarction 26 ; another treated 56 nonsaccular posterior circulation aneurysms with FD and observed only one death. 18 These improvements have been attributed to selecting patients who present early without established infarction on magnetic resonance imaging, strict dual antiplatelet regimens, and use of fewer, longer (35 mm) PED constructs. 18 26

In this analysis, the overall stroke rate after FD was 11.2% (8 of 71 patients), lower than previously reported rates of 17–23% 19 22 27 and did not differ from SAC. Known mechanisms of ischemic injury following FD include in-stent thrombosis, perforator occlusion, and jailed vessel occlusion. The last remains a theoretical risk without demonstrated clinical significance. 18 26 28 A prior meta-analysis estimated the rate of perforator occlusion at 3% with increased posterior circulation risk, 29 though recent angiographic series observed no perforator occlusion on immediate or follow-up imaging. 30 31 32 Though this study does not appreciate an increased ischemic risk with FD, careful selection of candidates for FD does seem to mitigate the risk of clinically significant ischemic stroke even if there is an increased incidence of stent thrombosis.

### Aneurysm Occlusion and the Role of Reconstructive Approaches

FD achieved satisfactory occlusion in 90.6% of patients (48/53) and a complete occlusion in 90% (36/40), a marked improvement over historical rates of 0–52% for giant and fusiform posterior circulation morphologies 20 26 33 and consistent with contemporary series reporting 75–100%. 18 26 33 Pooled 5-year data across 11 studies report a complete occlusion rate of 96%. 32 These improving outcomes with both reconstructive modalities reflect maturation in patient selection, device technology, and operator experience. 20 26 28 29 33 34

### Clinical Application

For fusiform and giant aneurysms where parent artery occlusion is contraindicated by inadequate collaterals or involvement of the basilar artery, dominant vertebral artery, distal posterior inferior cerebellar artery, anterior inferior cerebellar artery, or superior cerebellar artery, 2 a reconstructive approach with either FD or SAC is a promising option. However, this choice itself is conditional in many cases. SAC not only requires a saccular component to pack but also lacks the vessel reconstruction properties FD can provide in giant fusiform aneurysms. At present, the evidence supports equipoise across both modalities where both are technically feasible. In these cases, an individualized treatment approach is warranted, driven by aneurysm morphology, location, collateral adequacy, and patient-specific factors including tolerance of prolonged antiplatelet therapy. Further, the learning curve of new endovascular techniques should also not be underestimated, 35 especially in respect to FD as patient selection and operative experience continues to improve.

### Limitations

This analysis is limited by five nonrandomized, retrospective studies with inherent selection bias and limited statistical power. Morphological classification excluded dolichoectatic and dissecting subtypes, and aggregated outcomes precluded morphology-specific subgroup analysis (e.g., giant vs. fusiform). Functional outcomes were variably defined. Anatomical details critical to treatment selection (vertebral dominance, perforator anatomy, collateral sufficiency, prior infarct status) were inconsistently reported. Ischemic complications lacked mechanistic granularity, standardized imaging follow-up was absent, and no comparison to best medical therapy was available. Multi-institutional prospective studies are necessary to validate this study’s findings for nonsaccular posterior circulation aneurysms.

## Conclusion

Among patients with fusiform and giant posterior circulation aneurysms, FD and SAC produce comparable functional outcomes and occlusion rates. FD appeared to have higher all-cause mortality, aneurysm rupture, and in-stent thrombosis though these did not withstand sensitivity analyses and could not be separated from selection bias toward complex morphologies. Consideration between FD and SAC should continue to include anatomic territory, perforators, and tolerance of prolonged antiplatelet therapy.

## Declaration of GenAI Use

Generative AI (Claude, Anthropic) was used to assist with phrasing and manuscript organization. All scientific content, data interpretation, and conclusions are the authors’ own, and the authors take full responsibility for the final manuscript.

## Supplementary Material

Ergänzendes Material Supplementary Material
