# Supplementary material for: Endovascular Treatment of Nonsaccular Posterior Circulation Aneurysms: A Comparative Meta-analysis of Flow Diversion versus Stent-Assisted Coiling
Source: J Neurol Surg Rep. 2026 Oct 8;87(4):e183–91. doi: 10.1055/a-2964-8885 (PMC13649341; doi:10.1055/a-2964-8885)
Supplement: Supplementary file 1 [file 10-1055-a-2964-8885_29701418.pdf]

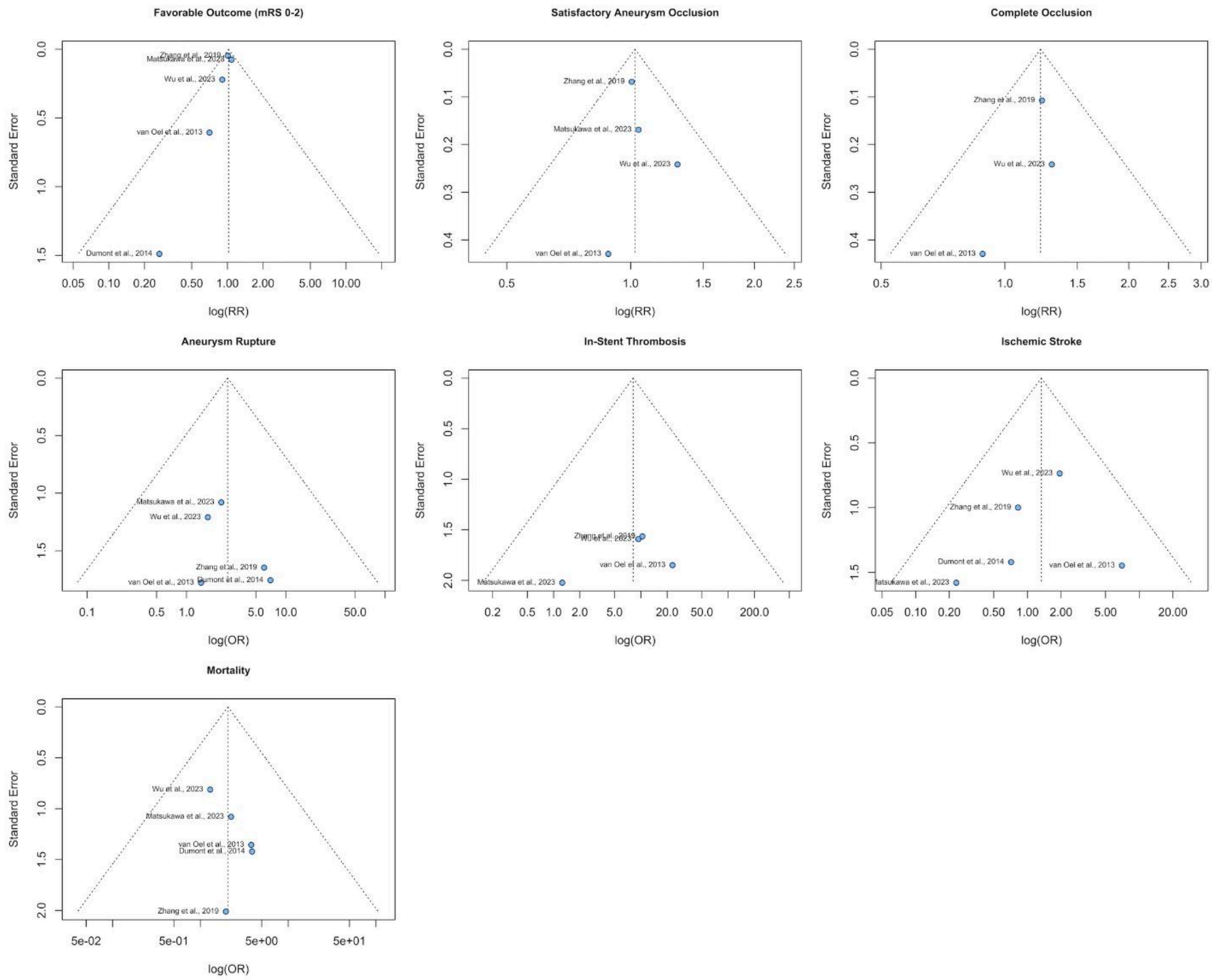

Supplementary Figure 1

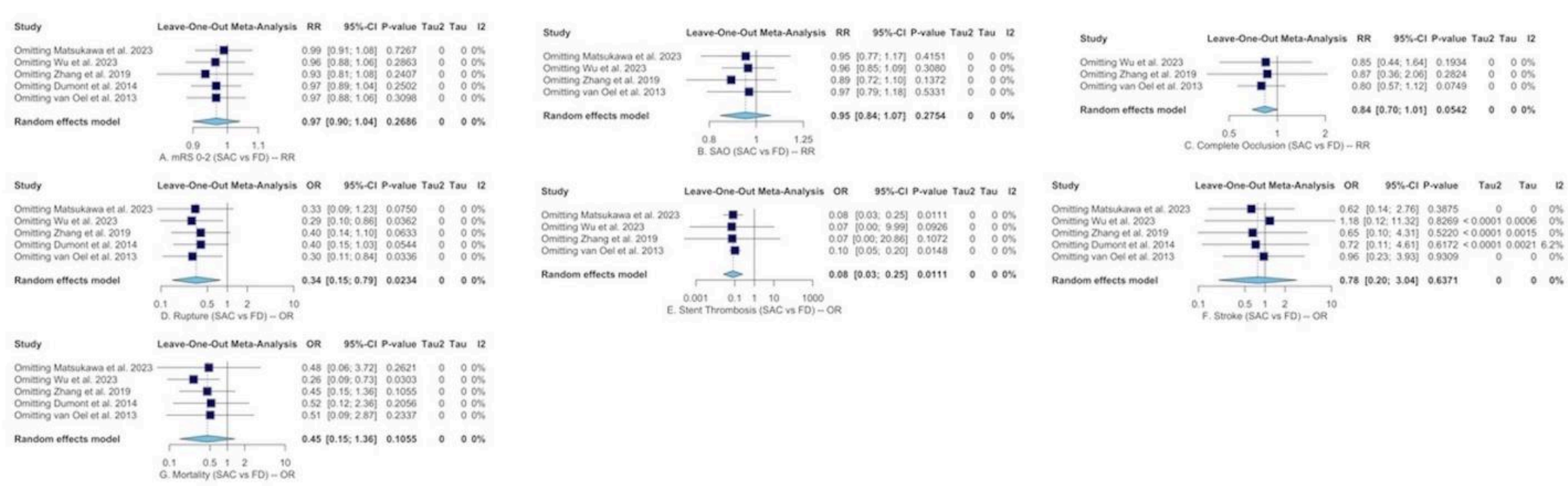

Supplementary Figure 2

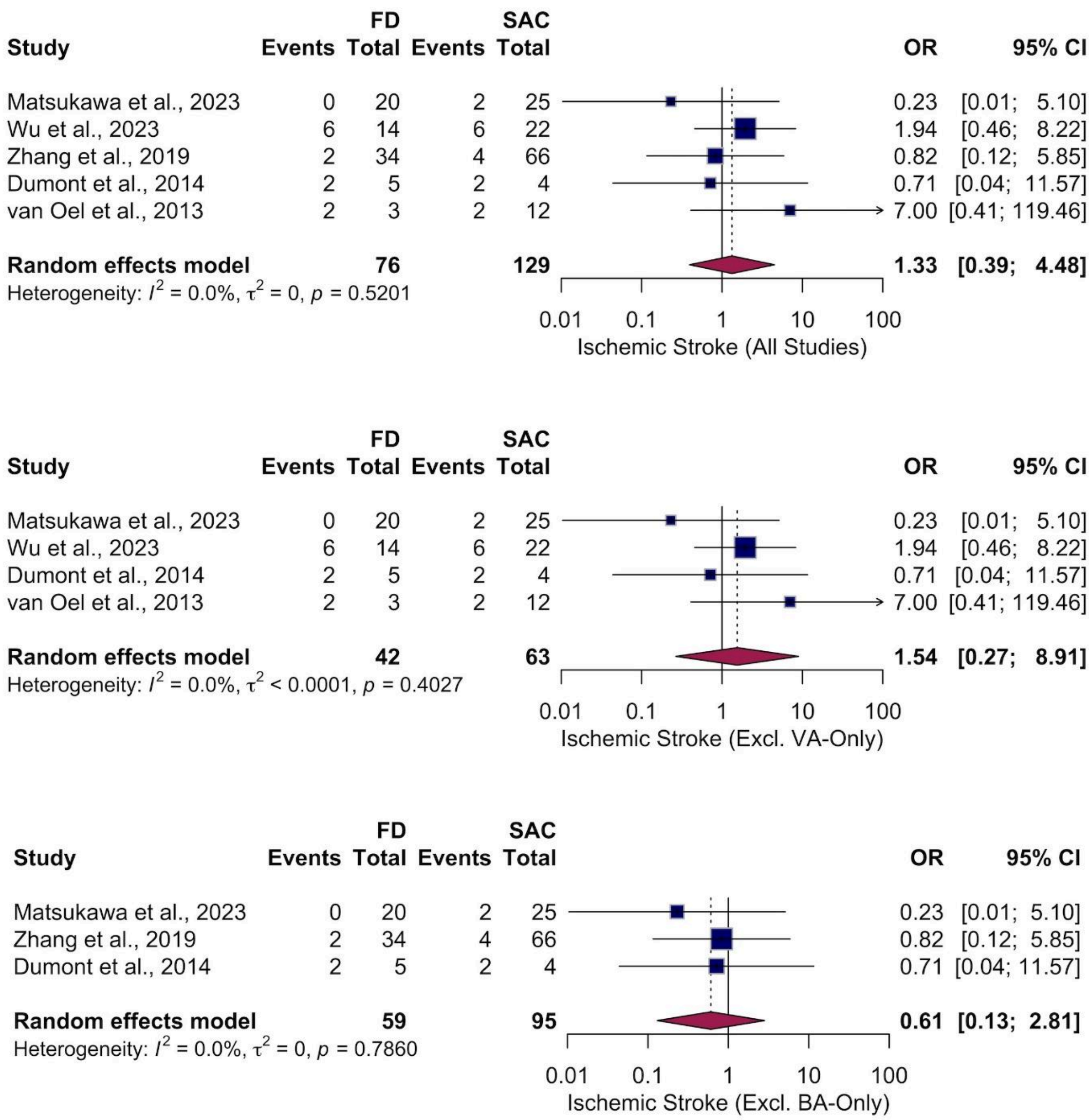

Supplementary Figure 3

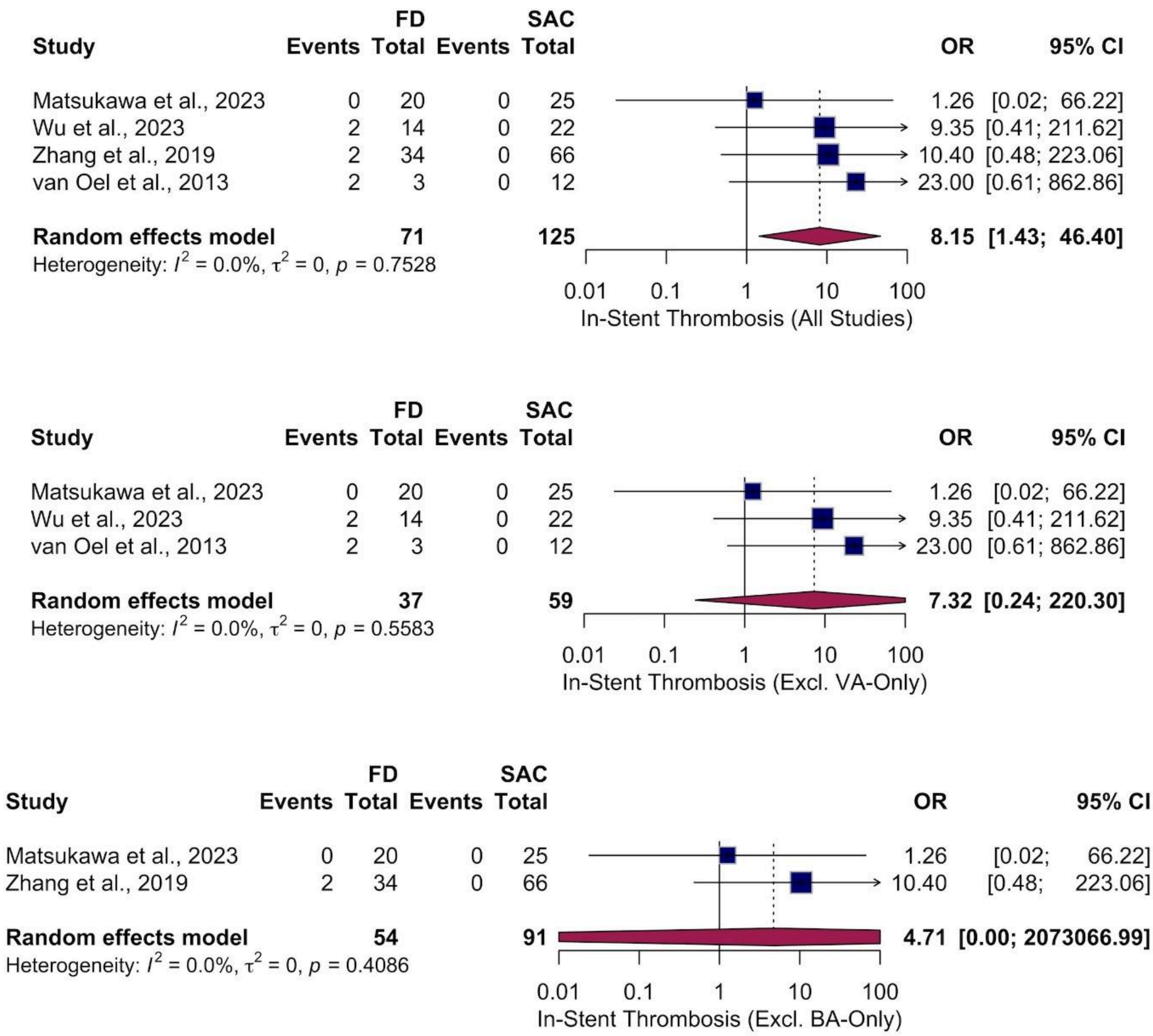

Supplementary Figure 4
